# Carbon Nanotube-Incorporated Nanofibers for Immunosensor Preparation against CD36

**DOI:** 10.1021/acsomega.2c07458

**Published:** 2023-01-30

**Authors:** Simge Er Zeybekler, Dilek Odaci

**Affiliations:** Biochemistry Department, Faculty of Science, Ege University, 35100Bornova-Izmir, Turkey

## Abstract

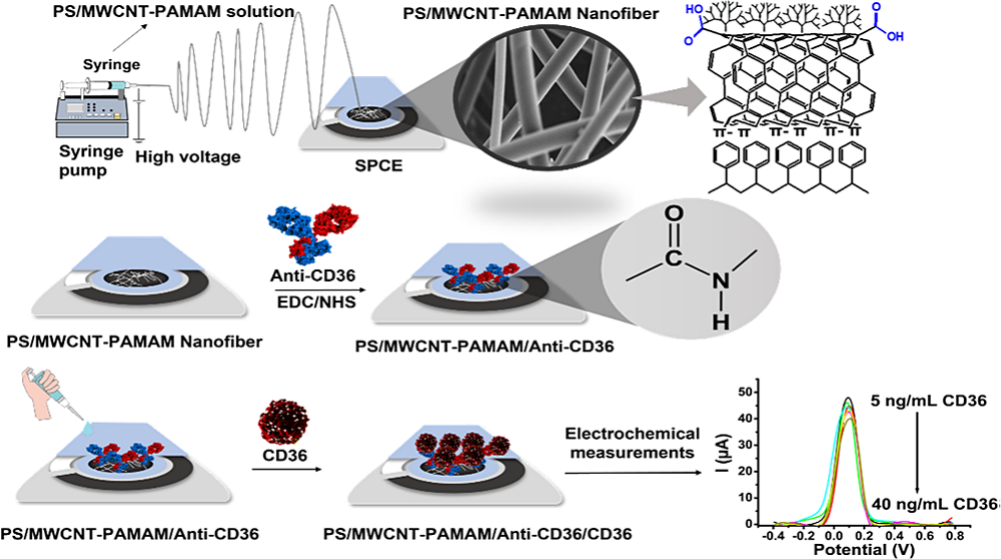

The increased serum concentration of CD36 is significantly associated with atherosclerosis, insulin resistance, and diabetes mellitus. Currently, there is no sensor system used for the detection of CD36 in the clinical field. Therefore, there is a need to develop a sensor system for the detection of CD36. The large surface area/volume ratio and controllable surface conformation of electrospun nanofibers (ENs) make them highly attractive for immunosensor applications. In the present study, PS/MWCNT-PAMAM ENs were produced and used as an immobilization matrix of Anti-CD36. Thus, the electrochemical behavior of the developed nanocomposite-based ENs and their usage potential were investigated for immunosensor applications. First, an oxidized multiwall carbon nanotube (MWCNT-OH) was synthesized and modified with a polyamidoamine generation 3 (PAMAM G3) dendrimer. The synthesized MWCNT-PAMAM nanocomposite was mixed with polystyrene (PS) solutions at different ratios to produce bead-free, smooth, and uniform PS/MWCNT-PAMAM ENs. PS/MWCNT-PAMAM ENs were accumulated on a screen-printed carbon electrode (SPCE) using the electrospinning technique. A biofunctional surface on the PS/MWCNT-PAMAM EN-coated SPCE was created using carbodiimide chemistry by covalent immobilization of Anti-CD36. The analytic performance characteristics of the developed PS/MWCNT-PAMAM/Anti-CD36 immunosensor were determined by performing electrochemical measurements in the presence of the CD36 protein. The linear detection range was found to be from 5 to 40 ng/mL, and the limit of detection was calculated as 3.94 ng/mL for CD36. The developed PS/MWCNT-PAMAM/Anti-CD36 immunosensor also displayed high tolerance to interference substances, good repeatability, and high recovery percent (recovery%) for artificial blood serum analysis.

## Introduction

Cardiovascular diseases, a general term used for such diseases including high blood pressure, atherosclerosis, and stroke, are among the deadliest diseases in the world. The relationship between the cardiovascular disease and diabetes has been well known for a long period of time.^1^ Atherosclerosis is defined as the obstruction and hardening and develops with the formation of cholesterol and plaques (fat deposits) on the inner wall of the arteries. Inflammation leads to the appearance of thrombotic complications of atherosclerosis. This event is completely related to the metabolism of carbohydrates and lipids. CD36 is an 88 kDa transmembrane glycoprotein that is interpreted as a biomarker for atherosclerosis, prediabetes, and type 2 diabetes mellitus (DM).^2^ It was reported that the serum concentration of CD36 is considerably correlated with atherosclerosis and insulin resistance, DM, and fatty liver markers and its serum concentration should be lesser than 25.3 ng/mL in nondiabetic plasma.^3,4^ Currently, there is no electrochemical sensor system used for the detection of CD36 in the clinical field commercially. Therefore, there is a need to develop a sensor system for the detection of CD36.

Nowadays, numerous studies have concentrated on improving devices that allow rapid detection and point-of-care analysis.^5^ Sensor systems attract a lot of attention due to their miniaturization properties, short analysis time, less sample volume requirement, and improved analytical properties.^6,7^ These analytical tools are still going on being upgraded continuously. Especially, label-free immunosensors have attracted a lot of attention since they offer high selectivity, specificity toward the targeted analyte, and low-cost diagnosis of biomolecules related to different diseases.^8^ Many nanomaterials such as carbon nanotubes (CNTs),^9^ graphene oxide (GO),^10^ colloidal nanoparticles,^11^ quantum dots,^12^ electrospun nanofibers (ENs),^13^ and dendrimers^14^ can be used in sensors. Among them, CNTs can provide direct electron transfer between the electrode and the receptor.^15^ Additionally, interest in ENs is increasing day by day due to their improved properties such as high adsorption capacity, porous structure, and biocompatibility.^16,17^ They are produced from polymer solutions under a high electric field by using the electrospinning technique. In addition, ENs can be produced by combining nanocomposites such as CNT^18^ and GO^19^ with polymer solutions.^20^ It is known that these nanocomposites have high electrical conductivity due to delocalized π-electrons in their benzene rings.^21^ Thus, nanocomposite-based ENs can exhibit advanced performance to produce desired immunosensor systems.

A polystyrene/graphene oxide-(3-aminopropyl)triethoxysilane/Anti-CD36 (PS/GO-APTES/Anti-CD36) immunosensor system was developed for the detection of CD36 using electrochemical measurements in our previous study, which was the first study of a label-free electrochemical immunosensor for CD36.^10^ This study is the second study of the label-free electrochemical immunosensor for CD36 detection in the literature. Thus, the electrochemical behaviors of the developed nanocomposite-based ENs and their usage potential were compared for immunosensor applications. In the present study, PS/MWCNT-PAMAM ENs were produced and used as an immobilization matrix of Anti-CD36. Although more than one technique such as wet, dry, melt, and gel spinning is used for nanofiber production, the electrospinning technique enables the mass production of continuous and longer nanofibers.^22^ Additionally, the electrospinning technique is considered a simple, low-cost method to produce nanostructures that are desired to be developed for sensor applications and create a suitable immobilization matrix for biological molecules.^16,23^ For this reason, the nanocomposite-based ENs were used to create a biofunctional surface on the working electrode using the electrospinning technique. A multiwall carbon nanotube (MWCNT) was used to produce ENs with high electrical conductivity and a fast electron transfer rate on the electrode surface. First, MWCNT-OH was synthesized and modified with a polyamidoamine generation 3 (PAMAM G3) dendrimer. It was aimed to form functional amine groups on the surface of nanofibers through PAMAM G3 dendrimer modification. Thus, a hydrophilic surface provided easier Anti-CD36 antibody immobilization. PS was chosen as the polymer because of its electrospinability and high molecular weight. Another reason for choosing PS was to form π–π interactions between MWCNT and PS. After the determination of the most suitable ratio of the MWCNT-PAMAM nanocomposite that should be added to the PS solution (35 wt %), PS/MWCNT-PAMAM nanofibers were deposited on the working electrode of a screen-printed carbon electrode (SPCE) using the electrospinning technique under the determined optimum conditions. Attenuated total reflection–Fourier transform infrared (ATR–FTIR) spectroscopy, scanning electron microscopy–energy-dispersive X-ray spectroscopy (SEM–EDS), X-ray photoelectron spectroscopy (XPS) techniques, and contact angle measurements were used for the physical and chemical characterizations of the synthesized PS/MWCNT-PAMAM nanofibers at each modification step. Covalent immobilization of Anti-CD36 on the PS/MWCNT-PAMAM nanofibers deposited on SPCE was carried out with *N*-(3-dimethylaminopropyl)-*N′*-ethylcarbodiimide hydrochloride/*N*-hydroxysuccinimide (EDC/NHS) carbodiimide chemistry to create a biofunctional surface on the SPCE. The analytic performance characteristics of the developed PS/MWCNT-PAMAM/Anti-CD36 immunosensor were determined by performing electrochemical measurements (differential pulse voltammetry (DPV), cyclic voltammetry (CV), and electrochemical impedance spectroscopy (EIS)) in the presence of the CD36 protein. Finally, the developed PS/MWCNT-PAMAM/Anti-CD36 immunosensor system also showed high tolerance to interference substances, good repeatability, and high recovery percent (recovery%) for artificial blood serum analysis.

## Experimental Methods

### Materials and Reagents

The CD36 monoclonal antibody (Anti-CD36, Ab-CD36, 185-1G2, 0.2 mg/mL) was purchased from Thermo Fisher. Recombinant human CD36 protein (His tag) (ab167735) was purchased from Abcam. PS (average *M*_w_ ∼ 350.000), MWCNT (>90%, *D* × *L*: 110–170 nm × 5–9 μm), PAMAM dendrimer (ethylenediamine core, generation 3.0 (G3) solution 20% (wt %) in methanol), d-glucose (GLC, ≥99.5%), urea (99.0–100.5%), insulin (INS), bovine serum albumin (BSA, ≥98.0%), potassium hexacyanoferrate(III) (HCF, K_4_[Fe(CN)_6_]), NHS, EDC, *N,N*-dimethylformamide (DMF), potassium chloride (KCl), and nitric acid (HNO_3_, ACS reagent, ≥90.0%) were purchased from Sigma-Aldrich. “Millipore Milli-Q Ultrapure Water System” was used for obtaining distilled water. All reagents were of analytical grade and were used as received without further purification.

An artificial blood serum which consists of 5.0 mM KCI, 5.0 mM CaCI_2_, 4.7 mM (d+)-glucose, 2.5 mM urea, 0.1% human serum albumin, and 145 mM NaCl was prepared according to the literature.^24^

### Equipment

XPS (Thermo Scientific K-Alpha, USA), ATR–FTIR (PerkinElmer Spectrum Two for a range of 4000 to 600 cm^–1^, USA), and EDS (Thermo Scientific Apreo S, USA) techniques were used for examining the MWCNT-OH and MWCNT-PAMAM nanocomposites. ENs were obtained using a NanoWeb Electrospun 103 (MaviTech, Turkey). A New Era pump system (USA) was used as a syringe pump. Contact angle measurements of the PS/MWCNT-PAMAM ENs were performed with the Attension Theta goniometer (USA) apparatus. The SEM technique (Thermo Scientific Apreo S, USA) was used for investigating the morphological structure of PS/MWCNT-PAMAM ENs. Also, ATR–FTIR and EDS analyses were performed to investigate the PS/MWCNT-PAMAM ENs. Electrochemical measurements (CV, DPV, and EIS) were performed using a PalmSens4 potentiostat (Palm Instruments Houten, Netherlands) with the SPCE (C110 carbon working electrode (4 mm diameter), an Ag reference electrode, and a carbon auxiliary electrode (Metrohm, Switzerland)).

### Preparation of MWCNT-OH

For the oxidation of MWCNT, 70 mg of MWCNT was weighed and sonicated in 70 mL of HNO_3_ for 1 h and then left to oxidize in a magnetic stirrer under reflux for 24 h at 100 °C.^25^ After 24 h, the obtained MWCNT-OH nanocomposite was precipitated at 13.000 rpm, washed twice with distilled water and once with ethanol, and then dried at 60 °C for 24 h.

### Modification of MWCNT-OH with the PAMAM G3 Dendrimer

EDC (150 mg) and NHS (100 mg) were added to 25 mL of an aqueous solution containing 50 mg of MWCNT-OH that had been presonicated for 1 h. The solution was sonicated for 5 min in an ice bath environment. Then, 2.5 mL of PAMAM G3 methanol solution was slowly added to the prepared suspension. After 24 h of reaction at 25 °C in a dark and continuous stirring environment, the MWCNT-PAMAM nanocomposite was obtained by precipitation at 13.000 rpm and washed four times with ultrapure water to remove unreacted PAMAM.^26^

### Preparation of PS/MWCNT-PAMAM Nanofibers

A PS/MWCNT-PAMAM nanocomposite solution was prepared with 35% (wt %) PS and 0.4% (wt %) MWCNT-PAMAM in DMF, and then the nanocomposite solution was stirred at 50 °C overnight. Then, 2.0 mL of the nanocomposite solution was placed into a syringe with a metal needle diameter of 8.8 mm. Electrospinning process was performed under the determined optimal conditions (applied voltage: 7.8 kV, tip-to-collector distance (TCD): 14 cm, flow rate: 1.2 mL/h, temperature: 26 °C, and humidity: 57%). The PS/MWCNT-PAMAM nanofibers were prevented from adhering to the surface of the reference and counter electrodes by punching round holes in the middle of the aluminum foil, equal to the diameter of the working electrode (4 mm diameter) during the electrospinning process. The optimum time to cover the surface of each working electrode that was attached at the same position in all experiments with an equal amount of nanofibers was determined as 4 min. PS/MWCNT-PAMAM-modified SPCEs were left to dry overnight at room temperature before performing covalent immobilization of Anti-CD36.

### Preparation of PS/MWCNT-PAMAM/Anti-CD36

EDC–NHS carbodiimide chemistry was used for covalent immobilization of Anti-CD36 to obtain a stable amide bond between the amine group of PAMAM and carboxyl groups of Anti-CD36. Next, 10 μg/mL Anti-CD36 was added to 100 μL of solution containing 500 mM EDC and 20 mM NHS solution in 10 mM phosphate-buffered saline (PBS) solution (pH 6.0) and left to incubate for 15 min at room temperature. Then, 5 μL of this mixture was dropped to the PS/MWCNT-PAMAM-modified electrode surface and incubated for 2 h at room temperature.

### Electrochemical Measurements

All measurements were carried out using SPCEs connected to a PalmSens4 instrument coupled to a computer (PSTrace 5.8 software). Electrochemical measurements (CV, DPV, and EIS) for bare SPCE, PS/MWCNT-PAMAM-modified SPCE, and PS/MWCNT-PAMAM/Anti-CD36-modified SPCE were performed at room temperature (25.0 ± 0.5 °C). CV and DPV measurements were performed at a scan rate of 50 mV/s between −0.4 and +0.8 V. The EIS trial was carried out in a frequency range of 0.21 × 10^–4^–100 kHz, an excitation voltage of 0.18 V, and a DC potential of 10 mV. All measurements were performed in 10 mM PBS (pH 7.4) containing 5.0 mM hexacyanoferrate(III) (HCF) as the redox probe and 0.1 M KCl.

### Data Analysis

ImageJ software was used to draw histogram graphs that indicate the nanofibers’ diameter distribution. The coefficient of variation (*cv*) is a statistical measure of how the standard deviation varies with the mean.^27^ The *cv* values of the nanofiber diameters were calculated using eq 1 to see how the nanofiber diameter averages changed according to the standard deviation.
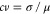
1where σ symbolizes the standard deviation (SD) and μ symbolizes the average diameter of fibers.^28^

## Results and Discussion

### Characterization of Functionalized MWCNT

**Figure 1:**
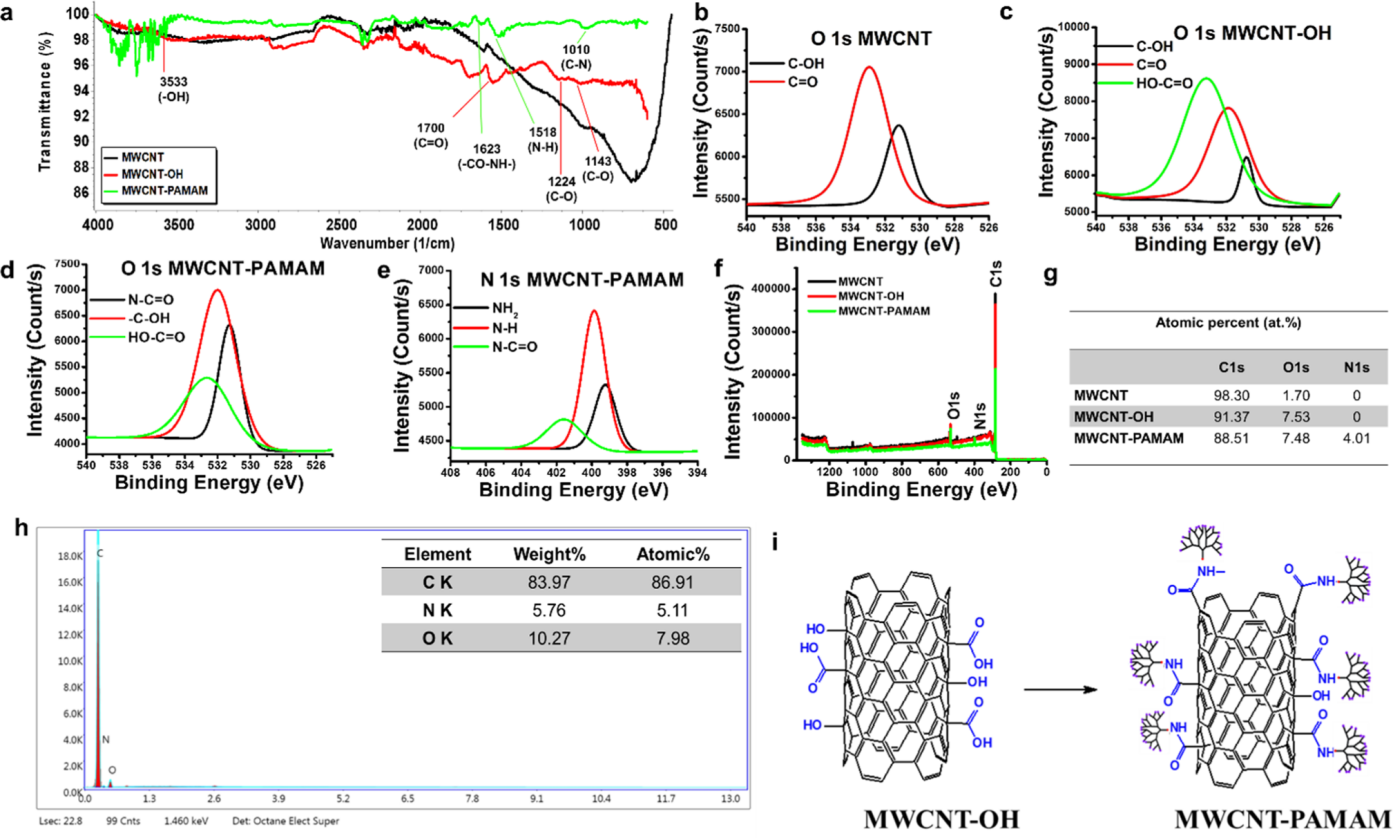
(a) ATR–FTIR spectra of MWCNT, MWCNT-OH, and MWCNT-PAMAM, (b) O 1s spectra of MWCNT, (c) O 1s spectra of MWCNT-OH, (d) O 1s spectra of MWCNT-PAMAM, (e) N 1s spectra of MWCNT-PAMAM, (f) XPS survey spectra of MWCNT, MWCNT-OH, and MWCNT-PAMAM, (g) chemical composition of MWCNT, MWCNT-OH, and MWCNT-PAMAM, (h) EDS spectrum of MWCNT-PAMAM, and (i) schematic representation of MWCNT-OH and MWCNT-PAMAM nanocomposites.

Characterization of the synthesized MWCNT-OH was investigated by ATR–FTIR and XPS techniques. There are no functional groups for covalent modification of biological molecules in pristine MWCNT due to sp^2^ hybridization between the carbon atoms. It can be seen that several functional groups such as hydroxyl and carboxyl are formed on the synthesized MWCNT-OH surface after the oxidation of MWCNT from Figure 1a. Specific peaks of MWCNT-OH bands at 3533 cm^–**1**^ (OH stretching of carboxyl groups), 1700 cm^–**1**^ (C=O stretching of carboxyl groups), 1143, and 1224 cm^–**1**^ (C–O stretching vibrations of the carboxylic acid group) were observed.^29,30^ All these obtained specific bands indicate that oxidation of MWCNT was successful. After modification of the MWCNT-OH nanocomposite with PAMAM G3, the characteristic peaks of MWCNT-PAMAM were observed at 1623 cm^–**1**^ (amide (−CO–NH−) bond), 1518 cm^–**1**^ (bending vibration of the N–H bond), and 1010 cm^–**1**^ (C–N bond).^31−33^ The decline of the band strength of the carbonyl group (C=O) at 1700 cm^–**1**^ compared to the band intensity of the ATR–FTIR spectra of MWCNT-OH is another evidence of the successful covalent modification of MWCNT-OH with the targeted PAMAM G3 dendrimer. All these specific bands indicate the successful covalent modification of MWCNT-OH with PAMAM G3.

XPS is a surface chemical analysis technique that offers beneficial information about the presence of functional groups and structural defects on the nanotube surface. Therefore, further characterization of the synthesized MWCNT-OH and MWCNT-PAMAM nanocomposites was carried out by the XPS technique. The peaks at 532.98 eV (C=O) and 531.08 eV (C–OH) seen in the O 1s spectrum of MWCNT confirm the presence of some carboxylic and hydroxyl groups on the surface of MWCNT (Figure 1b). After the oxidation of MWCNT, a carboxyl (HO=C–O) peak appeared at 533.38 eV, which was not seen in the MWCNT O 1s spectrum (Figure 1c).^34^ Three peaks were observed in the N 1s spectrum of MWCNT-PAMAM at 401.58, 399.88, and 399.28 eV. These peaks correspond to amide, secondary amine, and primary amine, respectively.^35^ Likewise, they are associated with the presence of an amide bond seen at 531.28 eV in the O 1s spectrum of MWCNT-PAMAM.^36^ The presence of the amide bond is indicative of the successful synthesis of MWCNT-PAMAM (Figure 1d,e). Furthermore, the main peak component in binding energy corresponds to approximately 285 eV in the C 1s spectrum and 534 eV in the O 1s spectrum, while the peak at 400 eV corresponds to N 1s in the wide survey scan spectrum (Figure 1f).^37^ A decrease was observed in the atomic percent of carbon (from 98.30 to 91.37%), while an increase was observed in the atomic percent of oxygen (from 1.70 to 7.53%) after oxidation of MWCNT. After the modification of PAMAM, the N 1s peak was observed, and its atomic percent was found to be 4.01% (Figure 1g). The EDS analysis was also performed to confirm that the MWCNT-PAMAM nanocomposite was successfully synthesized. Carbon (C), oxygen (O), and nitrogen (N) peak intensities were determined from the EDS spectrum of MWCNT-PAMAM, and elemental composition percentages were determined as 86.91, 5.11, and 7.98%, respectively (Figure 1h). The detection of nitrogen presence on the surface of the elemental nanocomposite in the EDS analysis evidences that the MWCNT-PAMAM nanocomposite was successfully obtained.

### Characterization of PS/MWCNT-PAMAM ENs

### Factors affecting the electrospinning process are classified into three main groups: solution parameters (concentration, viscosity, and conductivity of the polymer solution), system parameters (the flow rate, inner diameter of the needle, voltage, and TCD), and environmental (humidity and temperature) parameters.^38^ All these parameters directly influence the production of smooth and bead-free ENs, so all were taken into account during the study. Based on the literature, DMF alone is the best solvent in the preparation of PS nanofibers in comparison to other solvents or solvent mixtures including tetrahydrofuran (THF),^39^ DMF/DCM (v:v, 1:3),^40^ and THF/DMF (v:v, 1:3).^41^ Thus, DMF was chosen as the solvent for PS, while 35% (wt %) PS concentration was chosen in accordance with our previous optimization study.^10^ The other electrospinning parameters such as voltage, TCD, and flow rate were optimized again after the mixing of the PS polymer solution and the MWCNT-PAMAM nanocomposite to obtain bead-free and uniform nanofibers.

**Figure 2:**
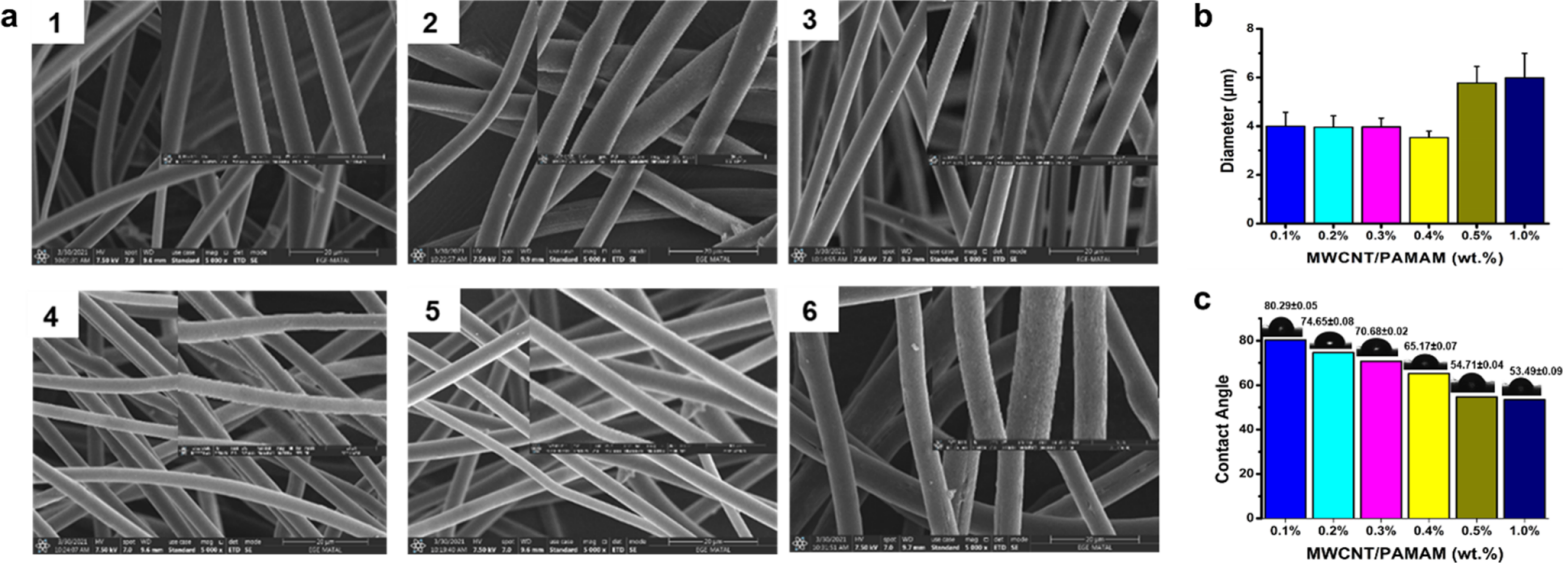
SEM images (5.000× magnification) of (a) PS/MWCNT-PAMAM 0.1% (wt %) (1), PS/MWCNT-PAMAM 0.2% (wt %) (2), PS/MWCNT-PAMAM 0.3% (wt %) (3), PS/MWCNT-PAMAM 0.4% (wt %) (4), PS/MWCNT-PAMAM 0.5% (wt %) (5), and PS/MWCNT-PAMAM 1.0% (wt %) (6) (magnifications of the inset SEM images are 25.000×), distribution of nanofiber diameter graphs of PS/MWCNT-PAMAM ENs (b), and contact angle measurements of PS/MWCNT-PAMAM ENs (c) (parameters of the electrospinning process: 7.8 kV, 14 cm TCD, and 1.2 mL/h flow rate).

Next, 0.1, 0.2, 0.3, 0.4, 0.5, and 1.0 (wt %) MWCNT-PAMAM was added to 35% PS (wt %) solutions to obtain PS/MWCNT-PAMAM nanofibers using the optimal electrospinning parameters. It was seen from the SEM images that bead-free nanofibers were obtained for all ratios (Figure 2a). Every SEM image was marked (70 different points) to analyze the distributions of the nanofiber diameters by using the ImageJ program. The calculated diameter distributions of PS/MWCNT-PAMAM nanofibers obtained with 0.1, 0.2, 0.3, 0.4, 0.5, and 1.0% (wt %) MWCNT-PAMAM ratios were found as 4.00 ± 0.57, 3.96 ± 0.47, 3.97 ± 0.36, 3.53 ± 0.27, 5.78 ± 0.88, and 5.99 ± 1.27 μm, respectively (Figure 2b). Detailed histogram graphs of the nanofiber diameter distributions at each MWCNT-PAMAM ratio are shown in Figure S1. The contact angle decreased (from 80.29 ± 0.05 to 53.49 ± 0.09) and the hydrophilicity of the surface increased owing to more amine groups formed on the surface with the increased ratio of the MWCNT-PAMAM nanocomposite (Figure 2c). The high surface hydrophilicity provides higher efficiency during the immobilization of biological molecules.^42^ Since the lowest cv and low contact angle values were seen in the nanofiber obtained with 0.4% (w/v) MWCNT-PAMAM ratio, 0.4% (wt %) MWCNT-PAMAM ratio was chosen to produce PS/MWCNT-PAMAM ENs.

**Figure 3:**
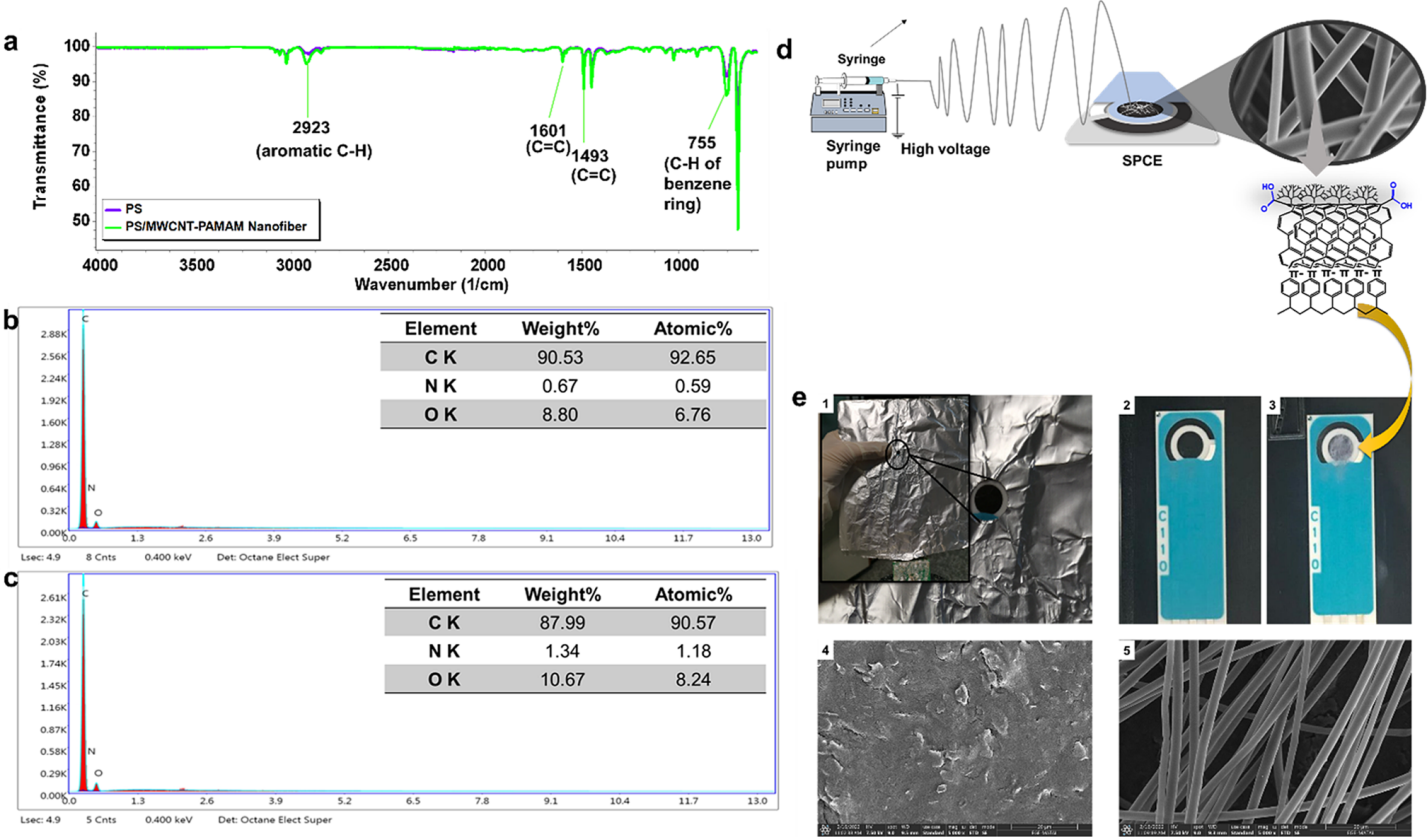
(a) ATR–FTIR spectra of 0.5% (wt %) PS/MWCNT-PAMAM ENs, EDS spectra of (b) 0.3% (wt %) PS/MWCNT-PAMAM, and (c) 0.5% (wt %) PS/MWCNT-PAMAM ENs, (d) schematic illustration of the production of PS/MWCNT-PAMAM EN-modified SPCE, and (e) (1) image of SPCE before the electrospinning process, (2) bare SPCE, (3) PS/MWCNT-PAMAM EN-modified SPCE after the electrospinning process, and SEM images (5.000× magnification) of (4) bare SPCE and (5) PS/MWCNT-PAMAM EN-modified SPCE.

The ATR–FTIR spectra of PS and 0.4% (wt %) PS/MWCNT-PAMAM ENs are shown in Figure 3a. In the ATR–FTIR spectra, only the characteristic peaks of PS were observed at 755 cm^–1^ (C–H vibration from the benzene ring), 1493 cm^–1^ (C=C stretching), 1601 cm^–1^ (aromatic C=C), and 2923 cm^–1^ (C–H stretching).^43^ The expected amine groups of PS/MWCNT-PAMAM ENs were not detected in the ATR–FTIR spectrum. It is thought that the high amount of PS used in nanofiber production suppressed the visibility of the specific bands of MWCNT-PAMAM. Thus, an EDS analysis was performed to detect the presence of nitrogen in 0.3 and 0.5% (wt %) PS/MWCNT-PAMAM ENs (Figure 3b,c). C, N, and O peak intensities were determined in the EDS spectrum of 0.3% (wt %) PS/MWCNT-PAMAM, and the elemental composition percentages were found as 92.65, 0.59, and 6.76%, respectively. The elemental composition percentages of C, N, and O in the EDS spectrum of 0.5% (w/v) PS/MWCNT-PAMAM are 90.57, 1.18, and 8.24%, respectively. The proportional increase in nitrogen content due to the increased MWCNT-PAMAM ratio (from 0.59 to 1.18%) is proof of the successful production of PS/MWCNT-PAMAM ENs.

### Electrochemical Characterization of PS/MWCNT-PAMAM/Anti-CD36

**Figure 4:**
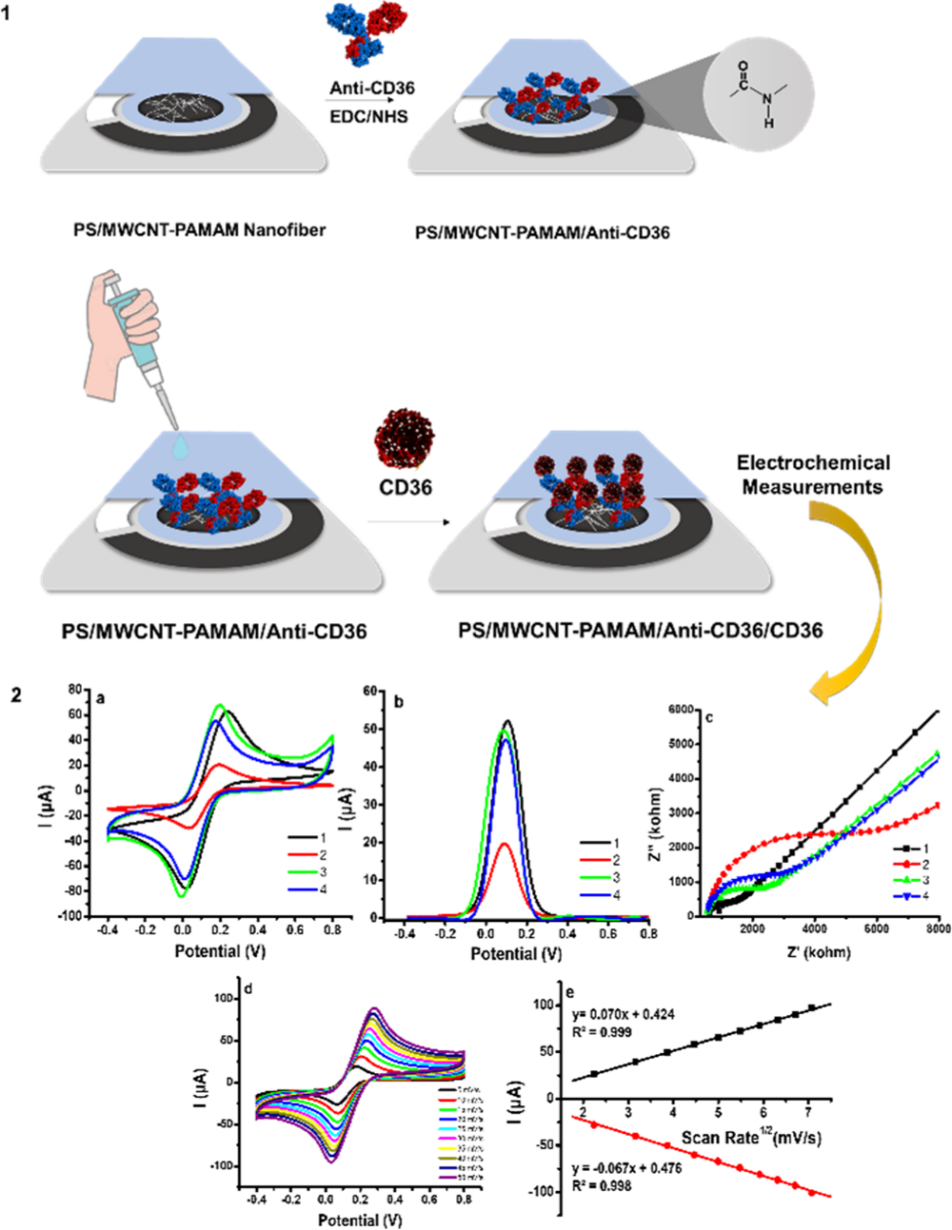
(1) Schematic illustration of the production of PS/MWCNT-PAMAM/Anti-CD36-modified SPCE and detection of CD36. (2a) CV profile of bare SPCE (1), PS/MWCNT-PAMAM (2), PS/MWCNT-PAMAM/Anti-CD36 (3), and PS/MWCNT-PAMAM/Anti-CD36/CD36 (4); (2b) DPV profile of bare SPCE (1), PS/MWCNT-PAMAM (2), PS/MWCNT-PAMAM/Anti-CD36 (3), and PS/MWCNT-PAMAM/Anti-CD36/CD36 (4); (2c) Nyquist profile of bare SPCE (1), PS/MWCNT-PAMAM (2), PS/MWCNT-PAMAM/Anti-CD36 (3), and PS/MWCNT-PAMAM/Anti-CD36/CD36 (4); (2d) CV curve of PS/MWCNT-PAMAM/Anti-CD36-modified SPCE at different scan rates; and (2e) correlation between the square root of the scan rates versus the peak currents (all measurements were carried out in 10 mL of PBS (pH 7.4) containing 5.0 mM HCF and 0.1 M KCl, [CD36] = 20 ng/mL, and [Anti-CD36] = 10 μg/mL).

CV, DPV, and EIS techniques were used for the electrochemical characterizations of bare, PS/MWCNT-PAMAM, and PS/MWCNT PAMAM/Anti-CD36-modified SPCE. PS/MWCNT-PAMAM ENs were deposited on SPCE by preventing from adhering to the surface of the reference and counter electrodes by punching round holes in the middle of the aluminum foil, equal to the diameter of the working electrode (4 mm) during the electrospinning process (Figure 3e). Schematic illustration of the production of PS/MWCNT-PAMAM EN-modified SPCE and CD36 detection is demonstrated in Figure 3d and Figure 4-1, respectively. For the deposition video of nanofibers on SPCE using the electrospinning technique, see the Supporting Information.

CV is a common electrochemical technique that provides information about the redox reaction of molecular species on the electrode surface.^44,45^ According to CV voltammograms, *I*_anodic_ values were found to be 62.49, 20.51, 67.79, and 55.02 μA for bare SPCE, PS/MWCNT-PAMAM, PS/MWCNT-PAMAM/Anti-CD36, and PS/MWCNT-PAMAM/Anti-CD36/CD36-modified SPCEs, respectively (Figure 4-2a). Oxidation and reduction peaks of HCF were obtained in all of the CV profiles. Although there was a significant decrease in the current after the modification of the bare SPCE with the PS/MWCNT-PAMAM nanofiber, the current increased after the covalent immobilization of Anti-CD36 on the nanofiber surface. In the electrochemical measurements performed in the presence of CD36, a decrease in the current was observed with the binding of CD36 to the electrode surface due to the formation of an immunocomplex. The p*K*_a_ values of the tertiary amines in the inner parts of PAMAM dendrimers vary between 3 and 6, while the p*K*_a_ values of the primary amines in the outer parts vary between 7 and 9. Therefore, their biophysical properties are anticipated to change remarkably with the pH.^46^ Most of the amine groups of PAMAM dendrimers exist in the uncharged form in a pH ∼ 7.4 PBS medium. The p*K*_a_ value of the carboxyl groups remaining without reacting with PAMAM on the MWCNT-OH surface is 6.82.^47^ These functional groups that remain unreacted are expected to be negatively charged due to their p*K*_a_ values in pH ∼ 7.4 PBS medium. Since this negative surface charge electrostatically repulses HCF, the electron transfer rate decreased at the PS/MWCNT-PAMAM nanofiber interface. Hence, decreased current was observed in the CV profile after the working electrode was coated with the PS/MWCNT-PAMAM nanofiber. At the same time, the entrapment of the redox-active species in the HCF solution into the PS/MWCNT-PAMAM nanofiber is another factor that causes the reduction of the current response. This is an indication that the system exhibits thin-layer diffusion. An increased current was observed after electrochemical measurements were performed after the covalent binding of Anti-CD36 to the PS/MWCNT-PAMAM nanofiber-modified SPCE. Since antigen binding fragments (*F*_ab_)_2_ are generally positively charged in the PBS medium at pH 7.4, electrostatic attraction occurred between HCF and the PS/MWCNT-PAMAM/Anti-CD36-modified SPCE, which causes an increased electron transfer rate resulting in a higher electrochemical current.^48^ Ozoemena et al. developed an electrochemical immunosensor based on onion-like carbon–polyacrylonitrile fiber (OLC–PAN) hybrids for the detection of *Vibrio cholerae* toxin. They obtained decreased current after the deposition of OLC–PAN fibers on the working electrode. They indicated that this phenomenon was observed due to the porous electrode surface. After the covalent immobilization of Ab to the fiber-modified electrode surface, they obtained an increased current compared to the fiber-modified electrode.^49^ Therefore, the electrochemical profile we obtained in this study is supported by the literature.

DPV is a derivative of linear sweep and pulsed voltammetry. It is frequently used for quantitative analyte determination due to its low detection limit, reduced capacitive current, and low background properties. The current is measured before and after the pulse is applied and the difference is taken to reduce the share of capacitive current in the measured current and increase the selectivity. It allows peak maximums to be obtained even for substances with half-wave potentials as different as 0.04 to 0.05 V.^50,51^ Since DPV makes peak discrimination even in small wave ranges, this technique was used for quantitative CD36 detection in this study. Figure 4-2b shows the DPV profile of bare, PS/MWCNT-PAMAM, PS/MWCNT-PAMAM/Anti-CD36, and PS/MWCNT-PAMAM/Anti-CD36/CD36-modified SPCEs. Just like in the CV profile, decreased current was obtained in the DPV profile after the surface of SPCE was coated with the PS/MWCNT-PAMAM nanofiber, and an increased current was observed when Anti-CD36 was attached to the PS/MWCNT-PAMAM nanofiber-coated electrode surface. In this respect, all DPV profiles were found to be compatible with the CV profiles.

EIS is an important electrochemical technique that provides information about the reactions carried out at the electrode interface. The response of current is measured by applying a sinusoidal voltage in this technique. The Nyquist plot (faradaic impedance spectrum) provides information about the double-layer capacitance, electron transfer resistance, ohmic resistance of the electrolyte solution, and Warburg diffusion impedance.^52^ The semi-circle diameter formed in the Nyquist plot is inversely proportional to the electrical conductivity. Therefore, we can have information about how the charge-transfer resistance changes at each modification step via the semi-circle diameter. The antibody immobilized to the surface or the antibody–antigen immunocomplex formed on the electrode surface prevents the redox-active molecules from spreading on the electrode surface by forming an insulating layer. This creates a change in capacitance, causing a change in the EIS profile of the electrodes.^53^ An increased ion charge-transfer resistance (R_ct_) was observed after the deposition of the PS/MWCNT-PAMAM nanofiber on the bare SPCE in the Nyquist profile. A decreased R_ct_ was observed after the Anti-CD36 modification of the PS/MWCNT-PAMAM nanofiber. Then, an increased R_ct_ was observed again because of the formation of the immunocomplex of CD36 with Anti-CD36 on the electrode surface (Figure 4-2c). These results showed that Nyquist profiles were compatible with both CV and DPV profiles.

The CV profile obtained at increasing scan rates gives information about whether the occurred reaction is quasi-reversible or reversible on the electrode surface. The anodic peak (*I*_anodic_) shifting to a higher potential and the cathodic peak shifting (*I*_cathodic_) to a lower potential while the peak potential separation (Δ*E*_p_) increases gradually depending on the increased scanning speed in the obtained CV profiles is an indication that the redox reaction occurring on the electrode surface is quasi-reversible.^15^ A faster voltage sweep creates a larger concentration gradient for redox-active species near the electrode. Thus, an increased current is observed with an increasing scan rate.^54^ Based on all this information, we can say that the redox reaction occurring on the electrode surface is quasi-reversible when we examine the CV profiles that we obtained at increasing scan rates (0–50 mVs^–1^) (Figure 4-2d). In addition, the linear variation of the anodic and cathodic peak currents with the square root of the scanning rate shows that the redox reaction occurring on the electrode surface is diffusion-controlled.^44^ The equations obtained for *I*_anodic_ and *I*_cathodic_ are *y* = 0.070*x* + 0.424 (*R*^2^ = 0.999) and *y* = −0.067*x* + 0.476 (*R*^2^ = 0.999), respectively (Figure 4-2e). It can be said that PS/MWCNT-PAMAM/Anti-CD36 showed a diffusion-controlled behavior and each modification step performed on the electrode surface was successful when considering all the results of this study.

### Application of PS/MWCNT-PAMAM/Anti-CD36 for the Detection of CD36

**Figure 5:**
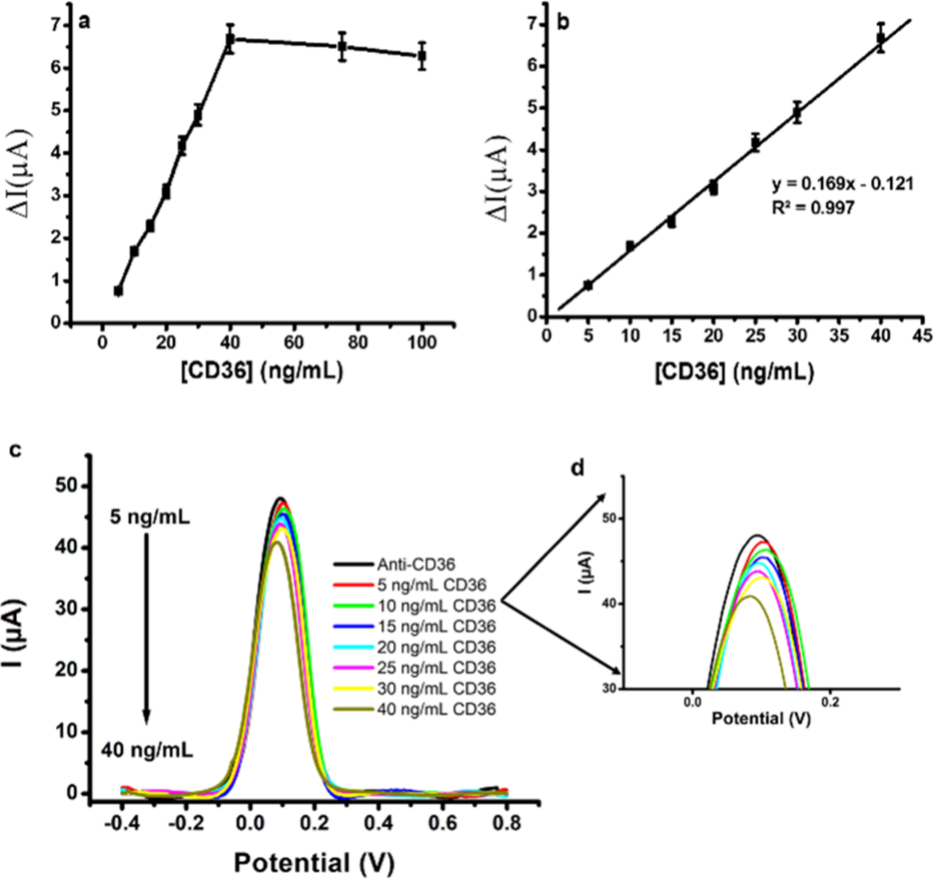
(a) Effect of the CD36 concentration on the immunosensor current response, (b) linear detection range for CD36 by the PS/MWCNT-PAMAM/Anti-CD36-modified SPCE, (c) DPV profile corresponding to increasing concentrations of CD36 between 5 and 40 ng/mL, and (d) magnified image of the current peaks (all measurements, scan rate: 50 mVs^–1^, in the presence of 10 mL of PBS (pH 7.4) solution containing 5 mM HCF and 0.1 M KCl, [Anti-CD36] = 10 μg/mL; error bars show the SB of at least 3 replicate measurements).

DPV measurements were performed at different concentrations of CD36 (5–100 ng/mL) to determine the linear detection range of CD36 by using the PS/MWCNT-/PAMAM/Anti-CD36 immunosensor system. The linear detection range of the PS/MWCNT-PAMAM/Anti-CD36 immunosensor was determined as 5–40 ng/mL. The equation of the calibration curve was determined as *y* = 0.169*x* – 0.121 (*R*^2^ = 0.997, *x* = CD36 concentration in ng/mL, and *y* = current in μA) (Figure 5b). It can be said that no considerable change in the current intensity was obtained after 40 ng/mL CD36 concentration owing to the steric hindrance of CD36. It has been indicated that the concentration of serum of the CD36 protein ought to be lower than 25.30 ng/mL for nondiabetic plasma in the literature.^4^Figure 5a shows the impact of the CD36 concentration on the immunosensor current response and the linear detection range for CD36 detection with the PS/MWCNT-PAMAM/Anti-CD36-modified SPCE. The linear detection range of the produced immunosensor was found as suitable for the detection of CD36 in blood serum.

Limit of detection (LOD) is defined as the lowest concentration of analyte that can be determined at a certain level of confidence.^55^ For this purpose, 10 different DPV measurements were performed with 5 ng/mL CD36 concentration, which is the lowest point of the calibration curve, to calculate the LOD of the PS/MWCNT-PAMAM/Anti-CD36 immunosensor. The SD and LOD were calculated using the 3SD/m formulation (SD: standard deviation of 10 measurement values performed at the lowest point of the calibration curve and m: slope of the calibration curve).^56^ As a result of all these calculations, the LOD of the PS/MWCNT-PAMAM/Anti-CD36 immunosensor was calculated as 3.94 ng/mL.

**Figure 6:**
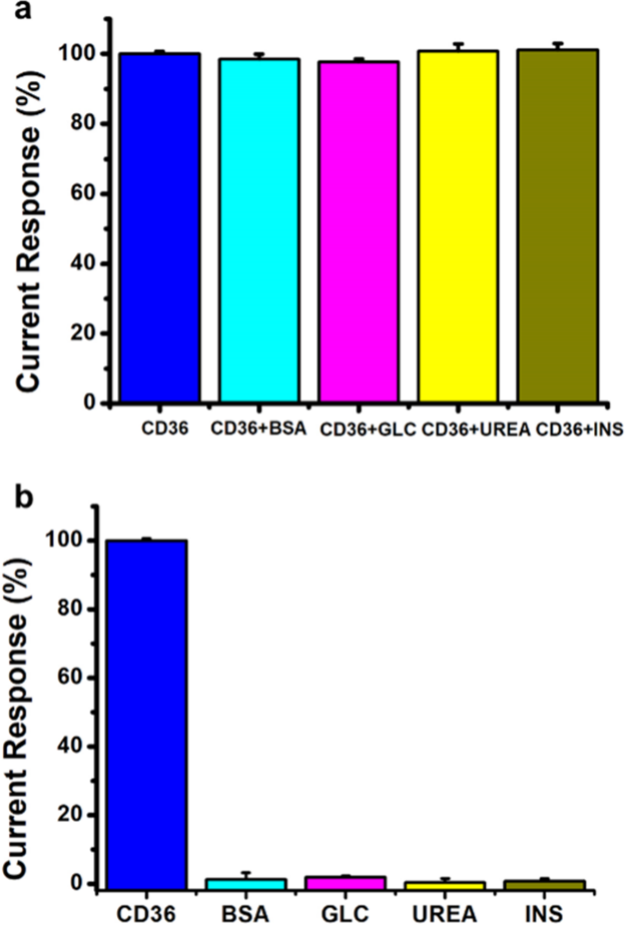
(a) Interference effect of BSA, GLC, urea, and INS on the detection of CD36 with the PS/MWCNT-PAMAM/Anti-CD36 immunosensor and (b) interference effect of BSA, GLC, urea, and INS alone on the PS/MWCNT-PAMAM/Anti-CD36 immunosensor response ([CD36]: 20 ng/mL, [BSA]: 4 g/dL, [GLC]: 100 mg/dL, [Urea]: 14 mg/dL, [INS]: 10 μU/mL, and [Anti-CD36]: 10 μg/mL) (all measurements, scan rate: 50 mVs^–1^, in the presence of 10 mL of PBS (pH 7.4) solution containing 5 mM HCF and 0.1 M KCl, [Anti-CD36] = 10 μg/mL; error bars show the SD of at least 3 replicate measurements).

The selectivity of an analytical method is determined by its ability to determine the analyte without being affected by other components in its matrix. Biological molecules in the blood serum that may interfere with the PS/MWCNT-PAMAM/Anti-CD36 immunosensor response were determined as BSA, GLC, INS, and urea. The reference ranges of BSA,^57^ GLC,^58^ INS,^59^ and urea^60^ were determined according to the literature. The selectivity of the developed PS/MWCNT-PAMAM/Anti-CD36 immunosensor was examined in the presence of 20 ng/mL CD36 solutions containing 4 g/dL BSA, 100 mg/dL glucose, 10 uU/mL INS, and 14 mg/dL urea through DPV measurements. It was determined that the selectivity was 98.56% for CD36 + BSA, 97.77% for CD36 + GLC, 101.22% for CD36 + INS, and 100.79% for CD36 + UREA. Selectivity studies were carried out for each interfering agent alone, too. The results obtained by evaluating all interfering agents alone were found to be 1.24% for BSA, 1.90% for GLC, 0.80% for INS, and 0.31% for UREA. It can be said that BSA, GLC, INS, and UREA with CD36 or alone did not have any interference effect on the developed PS/MWCNT-PAMAM/Anti-CD36 immunosensor response. The obtained results were calculated as relative. Figure 6a,b shows that the PS/MWCNT-PAMAM/Anti-CD36 immunosensor has high selectivity for CD36.^38^

**Figure 7:**
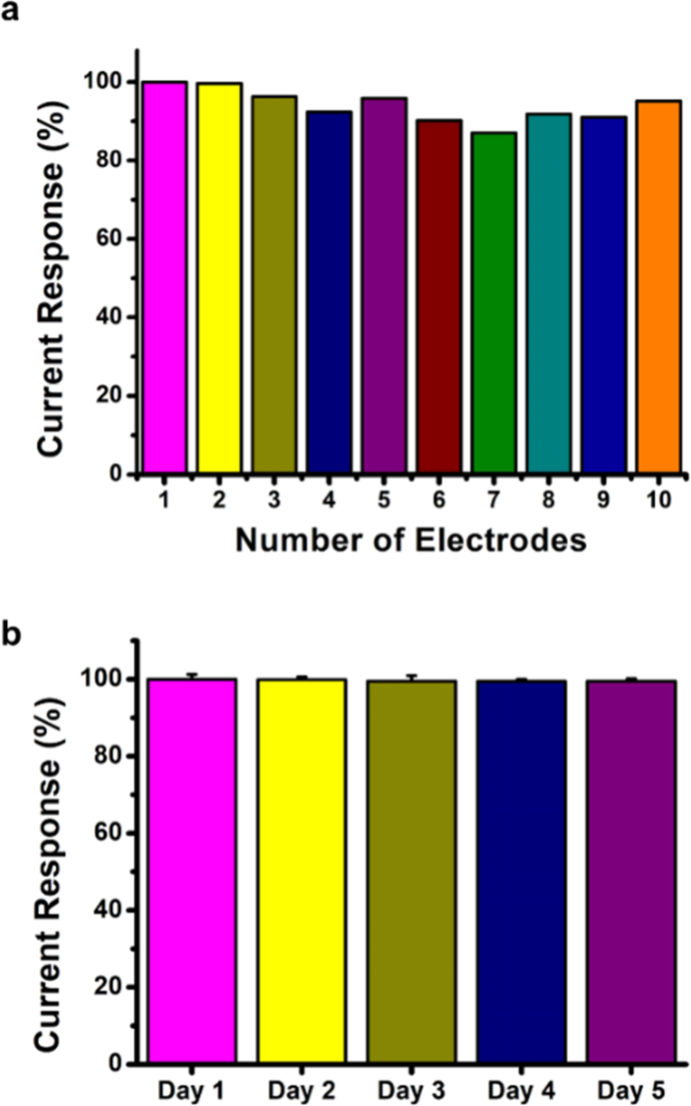
(a) Current response (%) for each prepared PS/MWCNT-PAMAM/Anti-CD36 immunosensor and (b) stability study. The PS/MWCNT-PAMAM/Anti-CD36 immunosensors were tested for 5 days of storage at 4 °C (all measurements, scan rate: 50 mVs^–1^, in the presence of 10 mL of PBS (pH 7.4) solution containing 5 mM HCF and 0.1 M KCl, [Anti-CD36] = 10 μg/mL, and [CD36] = 20 ng/mL).

Precision is an expression of how close the independent measurement results are to each other. Repeatability is the precision achieved under repeatability conditions.^61^ SD and *cv*% are used to express the precision of a set of repeated data. Both functions show how much the measured values deviate from the mean. Low *cv*% and SD values have notable significance as they define the reliability and repeatability of the immunosensors. For this purpose, the repeatability of the PS/MWCNT-PAMAM/Anti-CD36 immunosensor was evaluated by carrying out 10 separate DPV measurements in the presence of 20 ng/mL CD36 (Figure 7a). The *cv*% and SD values were calculated as 4.44% and ±0.13, respectively. Since the low value of *cv*% increases the precision of the method, a *cv*% value of less than 5% proves that the developed immunosensor offers us high precision.^50,62^ Stability studies were also performed to evaluate how to change the PS/MWCNT-PAMAM/Anti-CD36 immunosensor response when the immunosensor was stored at 4 °C for 5 days. The current response of the PS/MWCNT-PAMAM/Anti-CD36 immunosensors only had a change of 0.54% decrease according to the initial response after 5 days (Figure 7b). This indicates that the immobilized Anti-CD36 exhibits effective retention of the activity.

### Sample Application

The detection of CD36 in the blood serum is crucial since an increased CD36 concentration is significantly associated with not only atherosclerosis but also insulin resistance, DM and fatty liver markers. Recovery studies provide information on the accuracy of the system. According to the Eurachem guideline, the recovery study should be performed with at least 6 repetitions.^63^ For this reason, six PS/MWCNT-PAMAM/Anti-CD36 immunosensors were prepared separately. The recovery% was determined using a known amount of CD36 (20 ng/mL) added to the artificial blood serum and the concentration of CD36 measured with the PS/MWCNT-PAMAM/Anti-CD36 immunosensor. The result of recovery% performed with the artificial blood serum is shown in Table 1. The recovery% of CD36 was calculated as 97.66. Considering that the acceptable recovery percentages are 95–105%, this result confirms the validity of the system.^64,65^

**Table 1:** Recovery% of CD36 (ng/mL) in the Artificial Blood Serum with the PS/MWCNT-PAMAM/Anti-CD36 Immunosensor (*n* = 6)

artificial sample	added CD36	found CD36	*cv*%	recovery%
serum	20.00	19.53 ± 0.66	3.36	97.66

Except for our previous study, there is only one electrochemical sensor study about the detection of CD36 in the literature. Peng et al. produced a sandwich-type immunosensor for the detection of CD36 in human serum samples by using a modified cerium oxide nanohybrid loaded with prussian blue nanoparticles as a signal enhancer with glucose oxidase and modified this nanohybrid with Anti-CD36. After the modification of the electrode surface with chitosan–gold nanoparticle–His-tagged mouse monoclonal antibody–CD36, two developed nanomaterials were brought together, and electrochemical measurements were performed in the presence of glucose. The linear detection range of the sandwich-type immunosensor system was determined as 5.0 × 10^–3^–80.0 ng/mL, and the LOD was determined as 2 pg/mL.^65^ The detection limit of the developed immunosensor was found to be fairly low. However, integrating more than one biological molecule into the sensor reduces the stability of the system and its repeatability.^66^ Since biological materials such as antibodies and enzymes are easily affected by environmental conditions, their activity against the analyte may decrease.^67^ At the same time, steric hindrance can be created by occupying the adsorption sites in the labeled biosensor systems.^68^ In the present study, an immunosensor system was developed using only one biological molecule. Hence, lots of advantages were obtained such as a more stable system, low cost, and less time consumption for preparing electrodes. Although the PS/GO-APTES/Anti-CD36 immunosensor system, which was developed for CD36 detection in our previous study, exhibited a lower LOD for CD36 (0.99 ng/mL), it was found that both developed immunosensors have a suitable linear range to detect CD36 in the human blood serum successfully. Both studies have the feature of the first label-free electrochemical immunosensors developed for the detection of CD36 in the literature. We anticipate that the studies carried out in this context will fill the gap in the literature.

## Conclusions

In summary, we developed a label-free electrochemical immunosensor for the detection of CD36 based on a PS/MWCNT-PAMAM/Anti-CD36 modified SPCE. Additionally, PS/MWCNT-PAMAM ENs were produced and their electrochemical behaviors were investigated for the first time in the literature. CNTs tend to aggregate due to their inherent anisotropy. Moreover, the other difficulty of their usage is their very small sizes. Polymeric fibers obtained by mixing of CNTs with a polymer matrix enable us to obtain enhanced electrical conductivity and thinner smooth fibers.^69^ Moreover, the developed PS/MWCNT-PAMAM ENs can be used as an immobilization matrix for various biological molecules such as antibodies, enzymes, or aptamers. The PS/MWCNT-PAMAM/Anti-CD36 immunosensor exhibited a wide linear detection range for CD36 (5–40 ng/mL) and demonstrated a low LOD (3.94 ng/mL). In addition, the PS/MWCNT-PAMAM/Anti-CD36 immunosensor system showed high selectivity, repeatability, and recovery% (97.66%) in artificial blood serum. The only drawback of the developed PS/MWCNT-PAMAM/Anti-CD36 immunosensor is that it is disposable. However, the fact that it is nanocomposite-based and the system contains only one biological molecule reduces the cost considerably compared to ELISA kits. Considering all these results, it is thought that the developed immunosensor has application potential for rapid minimal-invasive detection of CD36, which is a biomarker for atherosclerosis, prediabetes, and DM in clinical analysis.

## Supporting Information Available

Video for electrospinning of PS/CNT/PAMAM/AntiCD36 on the SPCE (MP4)
Detailed graphs for the distribution of nanofiber diameter of PS/MWCNT-PAMAM ENs (PDF)
