# Supplementary material for: Carbon Nanotube-Incorporated Nanofibers for Immunosensor Preparation against CD36
Source: ACS Omega. 2023 Jan 30;8(6):5776–86. doi: 10.1021/acsomega.2c07458 (PMC9933220; doi:10.1021/acsomega.2c07458)
Supplement: Supplementary file 2 — ao2c07458_si_002.pdf [file ao2c07458_si_002.pdf]

Supporting Information

Carbon nanotubes incorporated nanofibers for immunosensor preparation against to CD36

Simge Er Zeybekler[a] and Dilek Odaci[a]*.

[a]Ege University Faculty of Science Biochemistry Department 35100 Bornova-Izmir/Turkey

KEYWORDS: Nanobiotechnology; nanotechnology; carbon nanotube; dendrimer; electrospun nanofiber; immunosensor.

Email: dilekodaci.od@gmail.com , dilek.odaci.demirkol@ege.edu.tr

Table of contents

1.	Detailed histogram graphics of nanofiber diameter distributions at each MWCNT–PAMAM ratios	S2

1. **Detailed histogram graphics of nanofiber diameter distributions at each MWCNT–PAMAM ratios**

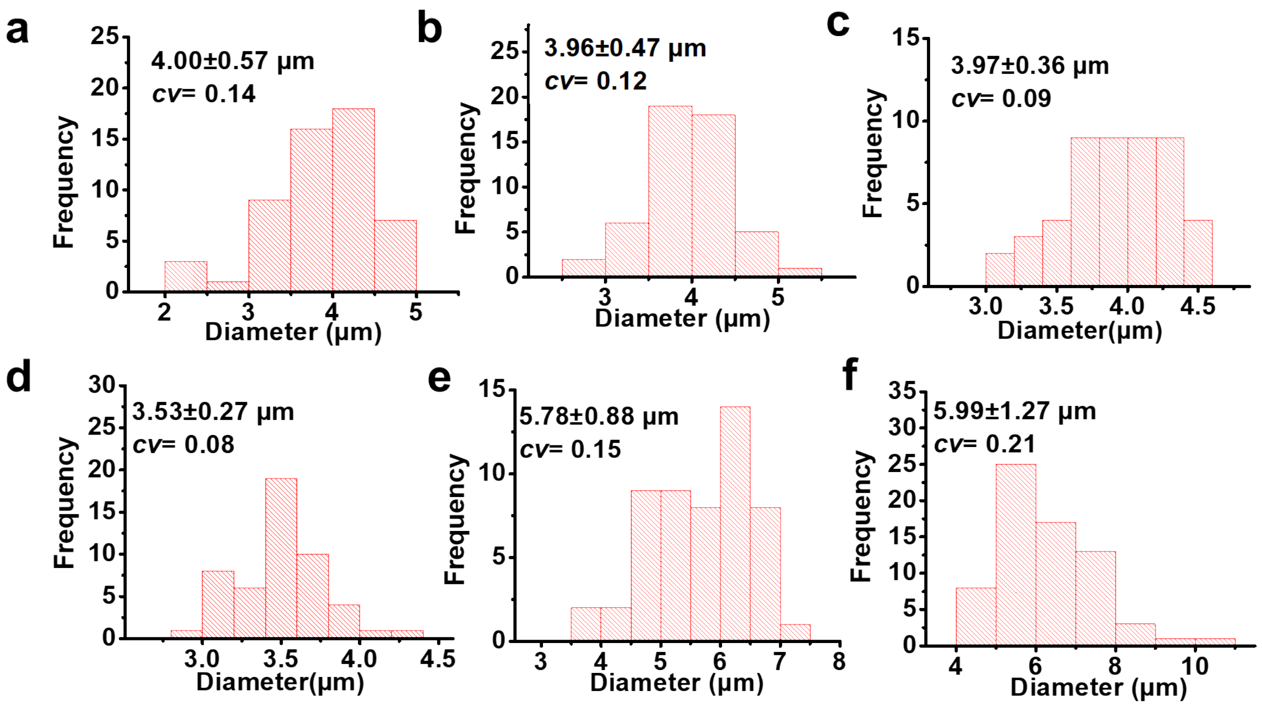

Figure S1. Graphs for the distribution of nanofiber diameter of PS/MWCNT-PAMAM ENs (a) PS/MWCNT-PAMAM 0.1% (wt.%), (b) PS/MWCNT-PAMAM 0.2% (wt.%), (c) PS/MWCNT-PAMAM 0.3% (wt.%), (d) PS/MWCNT-PAMAM 0.4% (wt.%), (e) PS/MWCNT-PAMAM 0.5% (wt.%), (f) PS/MWCNT-PAMAM 1.0% (wt.%).
